# Long-Term Outcomes Following Single-Stage Reamed Intramedullary Exchange Nailing in Apparently Aseptic Femoral Shaft Nonunion with Unsuspected Proof of Bacteria

**DOI:** 10.3390/jcm13051414

**Published:** 2024-02-29

**Authors:** Simon Hackl, Christian von Rüden, Katharina Trenkwalder, Lena Keppler, Christian Hierholzer, Mario Perl

**Affiliations:** 1 Department of Trauma Surgery, BG Unfallklinik Murnau, 82418 Murnau, Germany; 2 Institute for Biomechanics, Paracelsus Medical University, 5020 Salzburg, Austria; 3 Department of Trauma Surgery, Orthopedics and Hand Surgery, Weiden Medical Center, 92637 Weiden, Germany; 4 Institute for Biomechanics, BG Unfallklinik Murnau, 82418 Murnau, Germany; 5 Department of Trauma Surgery, University Hospital Zurich, 8091 Zurich, Switzerland; 6 Department of Trauma and Orthopedic Surgery, University Hospital Erlangen, Friedrich-Alexander University Erlangen-Nürnberg, 91054 Erlangen, Germany

**Keywords:** femur, fracture nonunion, outcome, septic, low-grade infection, intramedullary nail, SF-12, lower extremity functional scale (LEFS)

## Abstract

**Background:** The aim of this study was to evaluate detection rates and risk factors for unsuspected proof of bacteria, as well as clinical and radiologic outcomes following femoral shaft nonunion without clinical signs of infection treated by a single-stage surgical revision procedure including reamed intramedullary exchange nailing. **Methods:** A retrospective cohort study was performed in a European level I trauma center between January 2015 and December 2022. Fifty-eight patients were included who underwent reamed intramedullary exchange nailing as a single-step procedure for surgical revision of posttraumatic diaphyseal femoral nonunion without any indications of infection in medical history and without clinical signs of local infection. Clinical details of the patients were analyzed and functional and radiologic long-term outcomes were determined. **Results:** In all patients, with and without proof of bacteria osseous, healing could be observed. The physical component summary of the SF-12 demonstrated significantly better results at least one year after the final surgical revision in case of a negative bacterial culture during exchange nailing. **Conclusions:** Clinical long-term outcomes demonstrated a trend towards better results following femoral shaft nonunion revision if there was no evidence for the presence of low-grade infected nonunion. In this case, a single-stage surgical procedure may be recommended.

## 1. Introduction

Despite the ongoing development and optimization of surgical techniques and implants, impaired bone healing remains a challenging problem in fracture treatment, which is combined with a burden for the individual patient due to ongoing pain, as well as for society due to an enormous socioeconomic impact, such as therapy costs or productivity losses caused by relatively long treatment duration [1,2,3,4,5]. The reported prevalence of diaphyseal delayed union or nonunion of the femur reached up to 12.5%, mainly depending on the type of fracture stabilization [6,7]. It is a common consensus that the pathogenesis of nonunion is multifactorial and may be influenced, for example, by mechanical, metabolic and endocrine factors, as well as special medication such as non-steroidal anti-inflammatory drugs or the fracture pattern such as shaft fractures and the patient’s age [1,8,9,10,11,12]. In addition, the occurrence of infection at the fracture site is of significant importance in the pathogenesis of nonunion [13]. Despite the eye-catching appearance and the quite obvious diagnosis of an acute infection, chronic infection often could be characterized by a lack of clinical and laboratory signs of infection and is usually limited to the zone of the osseous lesion. Chronic infection also includes low-grade infection, which is mainly caused by low-virulence organisms with the ability of biofilm formation [14,15,16,17]. Thus, the development of nonunion could be the only symptom of low-grade infection. The diagnosis of low-grade infection is therefore much more difficult than that of acute infection. Microbiological and histological analyses of tissue samples collected from the nonunion area are the only appropriate way to differentiate between aseptic and septic nonunion caused by low-grade infection [18,19]. This is more critical since the treatment concepts and the surgical management of aseptic and septic nonunion are almost opposite: Reamed intramedullary exchange nailing as a single-step procedure is the treatment of choice for aseptic diaphyseal nonunion of the femur and is combined with a high rate of osseous union [20,21,22]. In the case of septic nonunion, the treatment concept is in accordance with the therapy principles of chronic fracture-related infection and involves a multi-step procedure, including debridement with removal of the implant and eradication of infection combined with antimicrobial therapy, subsequent revision osteosynthesis and reconstruction of the bone and soft tissue defect is performed [23,24,25,26,27,28]. Considering that the occurrence of low-grade infection is associated with the absence of clinical signs of infection, surgical revision of these cases is mainly performed as a single-step procedure without focusing on an accurate debridement, as it would be recommended in case of fracture-related infection since septic nonunion has not been recognized primarily [29]. Currently, the clinical impact of low-grade infection as an underlying cause of femoral shaft nonunion in regard to the surgical revision is unclear. Thus, the aim of this study was to evaluate detection rates and risk factors for unsuspected proof of bacteria, as well as the clinical and radiologic long-term outcome in a patient cohort with femoral shaft nonunion without clinical signs of acute infection who underwent single-stage surgical revision procedure with reamed intramedullary exchange nailing. Therefore, clinical details of the patients, as well as preoperative C-reactive protein (CRP) and white blood cell (WBC) counts, were analyzed, and functional and radiologic long-term outcomes were determined.

## 2. Materials and Methods

**Figure 1:**
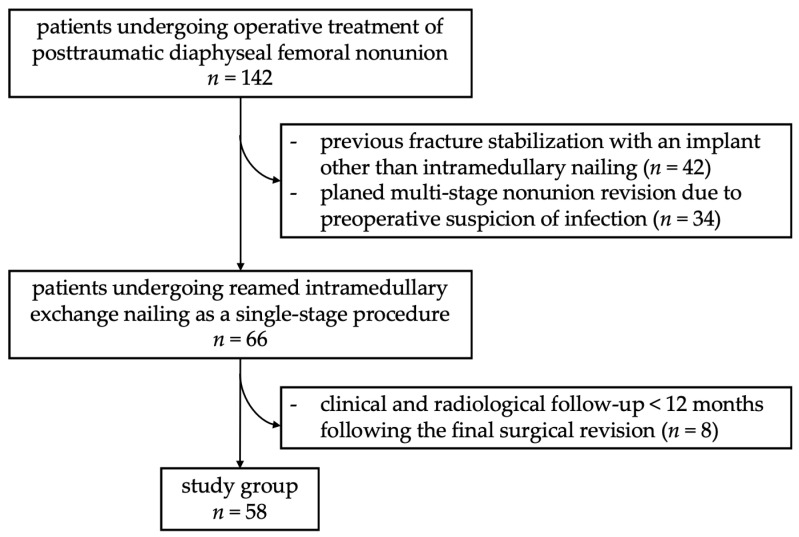
Overview of patients’ inclusion process.

A retrospective cohort study was performed in a European level I trauma center between January 2015 and December 2022. Fifty-eight patients were included who underwent reamed intramedullary exchange nailing as a single-step procedure for surgical revision of posttraumatic diaphyseal femoral nonunion without any indications for infection in medical history and without clinical signs of local infection, including pain at rest, redness, local hyperthermia, fever, persistent wound secretion and a sinus tract. If even a single parameter indicated a possible underlying infection, the patient was excluded from the study. In addition, patients treated with a surgical technique other than intramedullary nailing of a femoral shaft fracture were excluded from the study (Figure 1).

Clinical details of the patients are displayed in Table 1.

For the classification of the initial type of fracture, the AO/OTA classification was utilized. In the case of open fracture, the Gustilo–Anderson classification was used additionally [30]. The Carlson comorbidity index was used to objectify the morbidity of the study group [31]. Nonunion was defined clinically and radiologically after at least 6 months of missing osseous union during initial fracture treatment [32]. In 11 cases, diagnosis of nonunion was already made after 4 to 6 months due to a clear loss in progression of bone healing in regard to the current definition of the European Society of Tissue Regeneration in Orthopedics and Traumatology (ESTROT) [21,33]. Clinical signs of nonunion contained persistent instability in the fracture zone or inability to perform full weight bearing without pain. Radiographic evidence of nonunion was defined as the absence of osseous bridging in at least three of the four cortices as assessed on the antero-posterior and lateral views of conventional radiographs [34]. Whenever conventional radiographs were not conclusive enough to determine the diagnosis of nonunion, a computed tomography (CT) scan of the bony lesion was performed to clarify the presence of nonunion. Diagnosis of low-grade infection was made if at least two out of all four samples harvested during the surgical procedure demonstrated growth of bacteria in microbiological analysis and if clinical suggestive criteria for infection were missing [35].

### 2.1. Surgical Procedure

Surgical revision of diaphyseal femoral nonunion was performed in a standard manner and according to the diamond concept [36]. A preoperative single-shot microbiological prophylaxis using 1.5 g of cefuroxime was administered 30 min prior to the beginning of the surgical revision procedure. If contraindications concerning allergies existed, intravenous application of clindamycin was used. The patient was placed in a lateral position on a radiolucent operating table. The standard surgical procedure for diaphyseal femoral nonunion revision included the removal of the intramedullary nail used for initial fracture stabilization. Thereupon, a tissue sample on a dry swab (MASTASWAB, Mast Group Ltd., Bootle, UK), which was circulated 5 to 6 times around the part of the implant that had contact with the nonunion was gained for microbiological diagnostics. In the next step, a guide wire slightly bent at its tip was inserted into the femoral intramedullary canal and precisely positioned in the center–center position of the intercondylar region assessed by biplanar radiologic views. Then, stepwise reaming was carried out with the aim of osteogenic stimulus, as well as improving mechanical properties by inserting an intramedullary exchange nail with a larger diameter of at least 2 mm compared to the previous nail, plus a good cortical contact in the isthmus region and further microbiological diagnostics was performed using the initial graft material gained from intramedullary reaming [21,37,38]. For this purpose, one tissue sample with a swab that was circulated 5 to 6 times directly around the reaming graft material, and two tissue samples, each measuring at least 0.5 cm^3^ of the reaming graft material, were harvested [19]. In summary, four samples were obtained for microbiological diagnostics consisting of one tissue sample on a swab from the interface between the implant and nonunion and one tissue sample on a swab, as well as two tissue samples from the reaming graft material [39]. After ensuring that no gap or dehiscence was left at the fracture site, a T2 femur nail (Stryker Co., Ltd., Kalamazoo, MI, USA) with the option of interfragmentary compression was inserted to its correct position and the guide wire was removed. Then, distal interlocking screws were inserted and the femoral torsion was assessed: The femoral condyles were imaged in a lateral view with a precise projection of both condyles. The c-arm X-ray machine was then adjusted and moved in a strictly parallel direction until it was centered over the region of the femoral head. If the projection of the femoral head was anterior to the axis of the femoral shaft at two-thirds of its circumference, the femoral torsion was considered acceptable [40]. After compression was applied to the nonunion site, proximal interlocking was performed. Postoperatively, patients received physiotherapy with permitted weight bearing as tolerated. If low-grade infection—defined by at least two out of four samples demonstrating bacterial growth and without clinical indications for infection—was observed, test-specific and calculated antimicrobial medication was applied for at least six weeks after nonunion revision without any further surgical interventions. In case of postoperative clinical and laboratory signs of infection, removal of the intramedullary nail and a two- or multi-staged surgical procedure for eradication of infection was started [16].

### 2.2. Diagnostic Procedure

The tissue samples harvested on dry swabs during nonunion revision were immediately placed in the sterile swab container filled with protective Amies agar gel medium and were directly transferred to the on-site microbiological laboratory. These tissue samples were streaked out on Columbia agar with 5% sheep blood (bioMérieux, Hazelwood, MO, USA), Chocolat agar (PolyViteX, bioMérieux, Hazelwood, MO, USA), MacConkey agar (bioMérieux, Hazelwood, MO, USA) and thioglycolate broth (bioMérieux, Hazelwood, MO, USA). Samples were incubated in 5% CO_2_, as well as under anaerobic conditions at 37° Celsius for 48 h (short-term culturing). Morphologically distinct colony types were identified using a Vitek2 machine (bioMérieux Vitek Inc., Hazelwood, MO, USA) by MALDI-TOF mass spectrometry.

The tissue samples collected from the reaming graft material were directly inserted into a sterile containment prefilled with 9 mL of thioglycolate broth (bioMérieux, Hazelwood, MO, USA) and were immediately transferred to the on-site microbiological laboratory. After incubation in 5% CO_2_, as well as under anaerobic conditions at 37° Celsius for at least 14 days (long-term culturing), the suspension was additionally streaked out on Columbia agar with 5% sheep blood (bioMérieux, Hazelwood, MO, USA). Morphologically distinct colony types were identified as analogous to short-term culturing.

Laboratory values for systemic inflammation consisting of CRP concentrations and WBC counts were determined. These parameters were measured in peripheral blood samples drawn at the time point of hospital admission no more than two days before surgical nonunion revision [41]. Quantifications were performed by the institutional hematological laboratory during the regular preoperative diagnostic workup. The limit of determination for CRP concentration was <0.4 mg/dL and the cut-off value was determined at 1.0 mg/dL.

### 2.3. Follow-Up

After being discharged from the hospital, patients were clinically and radiologically followed up in the outpatient department at regular intervals: 6 weeks, 3 months, 6 months, and at least 1 year after the final surgical revision. The patients’ objective and subjective health status was assessed using the 12-item Short Form Survey (SF-12), which includes the mental component summary (MCS) and the physical component summary (PCS), as well as the Lower Extremity Functional Score (LEFS) [42,43].

### 2.4. Statistical Analysis and Ethical Standards

Statistical analysis was performed using IBM SPSS^®^ Statistics 26.0 for Windows (IBM Co., Ltd., Armonk, New York, NY, USA). The results of this study are presented as mean values ± standard deviation (SD) or median. Significance was statistically calculated based on the Mann–Whitney U test and Fisher’s exact test. Results were considered to be statistically significant with *p*-values < 0.05. G*Power 3.1 for Windows [44] was used to estimate the sample size. In regard to previous studies that compared the PCS of the SF-12 between femoral nonunion and normative group effect sizes (d) could be determined, which were between 1.35 and 2.55 [45,46,47]. Assuming the most unfavorable effect size (d) of 1.35, a sufficient power of 80% can be achieved with a sample size of 20 subjects and a probability of error (α) of 0.05. Written informed consent was given by all individuals participating in this study. The procedures involving human participants were in accordance with the bioethical standards of the institutional and national research committee (Bavarian Chamber of Physicians, ID 2017-162) and with the 1964 Helsinki Declaration and its following amendments.

## 3. Results

### 3.1. Rate of Low-Grade Infection in Femoral Shaft Nonunion

The study cohort consisted of 58 patients with apparently aseptic femoral shaft nonunion. Unsuspected proof of bacteria in at least two samples—followed by diagnosing low-grade infection—could be detected in the samples harvested during single-stage reamed intramedullary exchange nailing: in 10 cases (17%), positive bacterial cultures, meeting our criteria for low-grade infection, were detected following short-term culturing of the swabs and in 25 cases (43%) following long-term culturing of the tissue samples. The prevalence of cultured bacteria is presented in Table 2.

In 21 patients, a single organism was isolated from tissue samples harvested during intramedullary exchange nailing, whereas in 4 patients, a mixed culture with two different bacteria was detected. Only one polymicrobial culture was associated with an open fracture. Bacterial cultures remained negative in 48 cases (83%) following short-term culturing, whereas after long-term culturing, only 33 patients (57%) with apparently aseptic femoral shaft nonunion still had negative bacterial cultures.

The patient group with at least two surprising positive bacterial cultures with the same pathogen and no preoperative clinical signs of infection (group P) consisted of 21 male and 4 female patients with a mean age of 42.8 ± 3.3 (range 18–74) years. The group without proof of bacteria (group N) was composed of 24 male and 9 female patients with a mean age of 48.9 ± 2.8 (range 21–81) years (*p* = 0.162). The time internal between initial traumatic fracture treatment and surgical nonunion revision was 11.1 ± 1.6 (range 4–32; median 10) months in group P versus 11.2 ± 1.2 (range 4–25; median 8) months in group N (*p* = 0.951).

### 3.2. Evaluation of Risk Factors for the Occurrence of Positive Bacterial Cultures and/or Nonunion

In analyzing potential risk factors for the occurrence of positive bacterial cultures and/or nonunion, there was no statistical difference between both groups regarding the following parameters: Nicotine abuse was documented in eight cases in group P and in four cases in group N (*p* = 0.064). Three patients both in group P and group N were suffering from diabetes mellitus (*p* = 0.523). In addition, the Charlson comorbidity index was 0.32 ± 0.14 points in group P and 0.36 ± 0.14 points in group N (*p* = 0.831). In 20 of the 58 patients analyzed, a documented and anamnestic use of non-steroidal anti-inflammatory drugs could be observed, whereas in group P seven cases and in group N thirteen cases were recorded (*p* = 0.569). Regarding injury, as well as nonunion-related factors for the occurrence of positive bacterial cultures, despite a tendency with regard to the complexity of fracture pattern, only a significant difference could be found in regard to open soft tissue injuries. However, due to the small number of cases in this subgroup analysis, the relevance for clinical practice has to be used with caution (Table 3).

### 3.3. Preoperative Systemic Inflammation Markers

**Figure 2:**
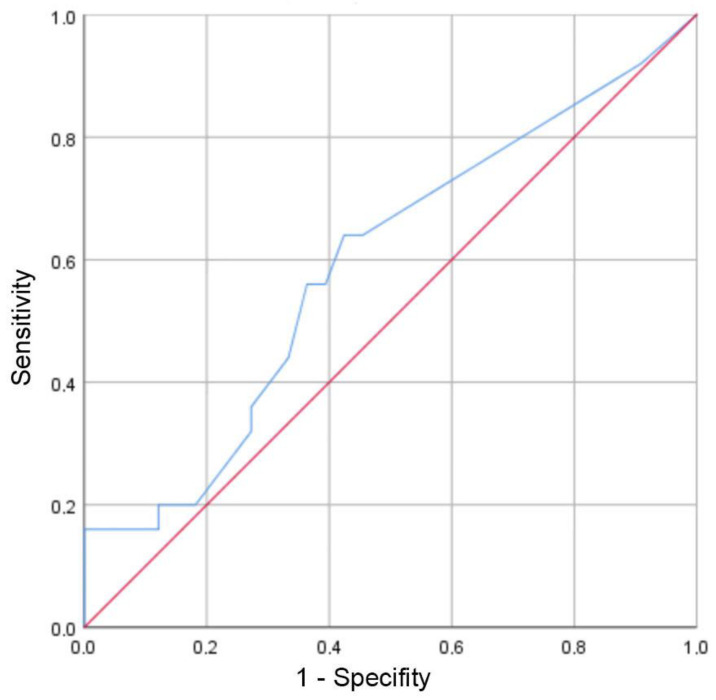
ROC curve of preoperative CRP values for diagnosis of positive bacterial culture. AUC = 0.591 (95% CI [0.441, 0.741]). Best possible cut-off value at CRP level of 0.6 mg/dL resulting in a sensitivity of 64% and a specificity of 58% (blue line: CRP; red line: reference line).

Patients in group P demonstrated a mean concentration of the preoperative CRP of 1.4 ± 0.3 (range 0.4–5.9; median 0.8) mg/dL and patients in group N of 0.8 ± 0.1 (range 0.4–3.3; median 0.4) mg/dL (*p* = 0.095). Considering patients with CRP levels above the cut-off value of 1.0 mg/dL, with 9 cases each in both groups, no statistically significant difference could be observed there, too (*p* = 0.477). Preoperative values for WBC of 8.0 ± 0.4 (range 4.6–12.4; median 8.1)/nL in group P and of 7.4 ± 0.4 (range 3.1–11.3; median 7.0)/nL in group N did not show a statistic significant difference (*p* = 0.249). In addition, the potential diagnostic efficiency of CRP level was analyzed by the receiver operating characteristic (ROC) curve with an area under the curve (AUC) of 0.591 (Figure 2).

A Youden index calculation demonstrated the best possible cut-off value at a CRP level of 0.6 mg/dL with a sensitivity of 64% and a specificity of 58%, demonstrating that no clinically relevant cut-off value could be observed in this patient cohort. With an AUC of 0.563 and the best possible cut-off value at a WBC level of 7.3/nL (sensitivity: 67%; specificity: 55%), this inflammatory marker was also not suitable for a clinically relevant prediction.

### 3.4. Objective and Subjective Outcome

**Figure 3:**
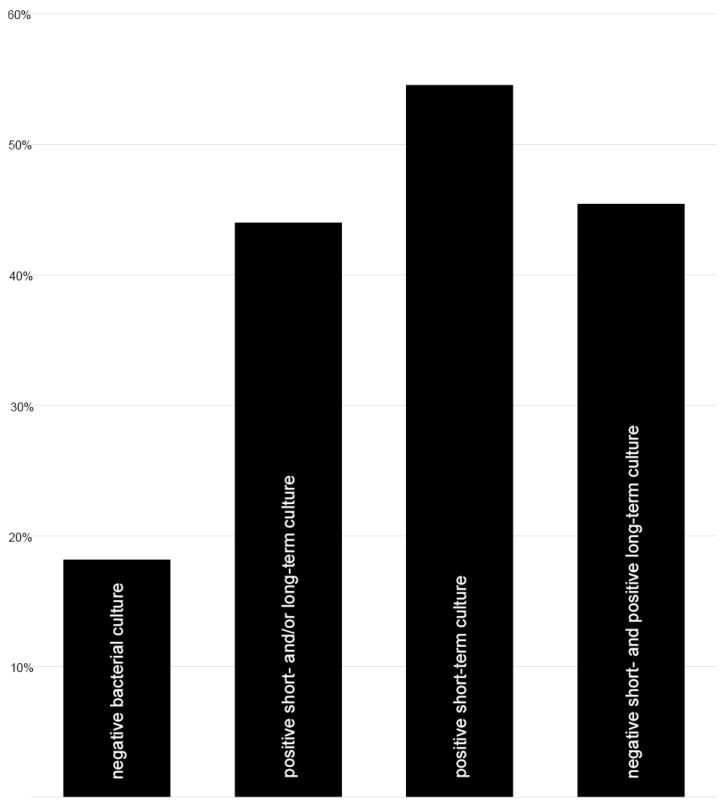
Patients with additional surgical interventions (%). Negative bacterial culture (n = 6 out of group N); positive short- and/or long-term culture (n = 11 out of group P); positive short-term culture (n = 6 out of 11 positive cultures); negative short- and positive long-term culture (n = 5 out of 11 positive cultures).

In all patients of both groups, a completed osseous healing could be observed. In group N osseous healing could be detected after 14.0 ± 2.0 (range 2–35; median 12) months and in group P after 15.3 ± 2.0 (range 2–32; median 17) months (*p* = 0.651). After exchange nailing in group N, 27 out of 33 patients (82%) healed without any further intervention, whereas 6 patients needed 1.3 ± 0.2 (range 1–2; median 1) additional surgical procedures to achieve osseous healing. In these patients, the following further procedures were performed: Three patients received a singular dynamization of the intramedullary nail, one patient received a further exchange nailing procedure to a larger diameter combined with bone grafting, and two patients underwent a dynamization of the intramedullary nail due to a lack of osseous healing repeating the exchanging nailing to a larger diameter nail, whereby in one of these two patients additional bone grafting was performed. In contrast, in group P, only 14 out of 25 patients (56%) healed after the exchange nailing procedure. However, none of these patients demonstrated fulminant systemic septic conditions after the exchange nailing procedure. Eleven patients needed 1.8 ± 0.2 (range 1–7; median 1) additional procedures for eradication of infection and achieving osseous healing. Hereby, the patients underwent the following further procedures: Seven patients received a debridement with a further exchange nailing procedure, three patients underwent debridement with the removal of the implant, followed by a further exchange nailing after negative bacterial cultures, and one patient received multiple debridements, followed by a further exchange nailing, due to ongoing delayed osseous healing dynamization of the intramedullary nail. In case of positive bacterial cultures and necessary additional surgical procedures, a collagen matrix loaded with either Gentamycin or Vancomycin was placed intramedullary—if one of these antibiotics was effective against the cultured microorganism. In summary, the different osseous healing rates in group N (82%) and in group P (56%) were statistically different (*p* = 0.032). Regarding the number of patients with additional further interventions, there was no significant difference in the positive bacterial growth that could be already detected after short-term culturing or only after long-term culturing (Figure 3).

In addition, regarding all data harvested, no clinically meaningful parameter could be found that leads to a statistically reliable statement if additional surgical procedures may be necessary following exchange nailing with unsuspected proof of bacteria. An example is provided here: CRP values in group P with additional surgical intervention were 1.5 ± 0.6 (range 0.4–5.9; median 0.6) mg/dl and CRP values in group P without additional surgical intervention were 1.3 ± 0.3 (range 0.4–4.5; median 0.9) mg/dL (*p* = 0.789); nonunion with initial open fractures in group P with additional surgical intervention were three cases and nonunion with initial open fractures in group P without additional surgical intervention were also three cases.

Regarding the objective outcome, represented by the LEFS, no statistically significant difference could be observed after the achievement of osseous healing in both groups. In contrast, the physical component summary of the SF-12, a display for the subjective outcome, demonstrated significantly better results at least one year after the final surgical revision in case of a negative bacterial culture during femur exchange nailing (Table 4).

Nevertheless, there was no statistically significant difference between patients without any further intervention (PCS of SF-12 42.3 ± 2.3 points) and those with additional surgical interventions (PCS of SF-12 35.9 ± 4.5 points; *p* = 0.205), regardless of whether there was proof of bacteria or not.

## 4. Discussion

Nonunion is defined as the failure of the bone to unite after the occurrence of a bone lesion that will not heal without further intervention, regardless of the length of treatment [32,48]. Despite the clinical appearance, 43% of the primarily aseptic categorized diaphyseal femur nonunion demonstrated positive bacterial cultures from intraoperative samples harvested during revision surgery, emphasizing the clinical relevance of low-grade infection. Although there are no acute clinical signs of infection, in almost every second patient with detection of bacterial growth additional surgical interventions are needed until osseous healing is reached, in contrast to only 20% of patients with negative bacterial cultures after single-stage reamed intramedullary exchange nailing.

Taking into account the period of time elapsed during nonunion development, it can be assumed that the infection responsible for the development of nonunion might potentially be chronic. Therefore, low-virulent bacteria including a mature biofilm on the fixation material must be assumed, which is in accordance with our findings of 21 coagulase-negative *Staphylococcus* spp. (CoNS) isolated from the total number of 29 bacterial isolates, as well as with other studies [49,50]. Thus, the basic principle in the treatment of chronic fracture-related infection with consistent removal of avital tissue and exchange of fixation material should be applied to septic femoral shaft nonunion [51,52]. Due to the insufficient addressing of the biomechanics that may underlie nonunion, implant retention is not expedient [17]. These principles are basically integrated into the single-stage reamed intramedullary exchange nailing, emphasizing the need to remove the previous osteosynthesis material [53,54] and aim for infection eradication to achieve nonunion healing, in combination with the avoidance of infection recurrence in the sense of chronic osteomyelitis after osseous healing, and, finally, the recovery of a sufficient regaining of function [55]. Nevertheless, the higher number of additional surgical revisions in case of positive bacterial culture necessary until osseous healing demonstrated in this study—44% of the femoral shaft nonunion with and 18% without proof of bacteria—is in accordance with the current multidisciplinary surgical treatment principles for septic diaphyseal femoral nonunion and could be also demonstrated by other studies analyzing nonunion at different locations, observing a revision rate in case of infected nonunion between 6 to 22% [56]. However, it is important to note that the final healing rates are similarly high.

On the other hand, 56% of diaphyseal femoral nonunion with unsuspected proof of bacteria healed after single-stage reamed intramedullary exchange nailing—in addition to test-specific antibiotic therapy—without any further intervention, which is, for example, comparable to diaphyseal tibial nonunion caused by low-grade infection [14]. These findings are in contrast to a study performed by Amorosa et al. analyzing the outcome of a single-stage treatment protocol for presumptive aseptic diaphyseal nonunion—including 87 cases of clavicular, humeral, radial, ulnar, femoral and tibial nonunion within 28.7% of the cases positive bacterial cultures—with a healing rate of 72% in cases of positive bacterial cultures and 93.6% in patients without proof of bacteria. However, no further information was given regarding the microbiological diagnostics, and also patients with at least one positive intraoperative culture were classified as infected [50]. In addition, the definition of nonunion varies widely in the literature, making it even more difficult to compare different studies [32]. Nevertheless, comparable results with a healing rate of 78% in cases of positive bacterial culture in presumed aseptic diaphyseal nonunion could be achieved by a single-stage surgical protocol—including nonunion revisions both with plate and nail fixation—described by Arsoy et al. [49].

In general, sufficient treatment of femoral shaft nonunion is a challenge for every trauma surgeon. The distinction between presumed aseptic and septic nonunion yields an additional complicating component in this regard. A tendency to develop septic nonunion was found with respect to the complexity of the fracture pattern, but the only significant risk factor for infection was an open fracture. This is in line with the known literature [51,57,58]. In addition, regarding all data harvested in the current study, no clinically meaningful parameter could be found that leads to a statistically reliable statement if additional surgical procedures may be necessary following exchange nailing with unsuspected proof of bacteria.

The development of septic nonunion occurs in two ways: On the one hand, an early infection can develop into manifest infectious nonunion if not treated optimally with antimicrobial agents alone. On the other hand, a pathogen of relatively low virulence can cause a low-grade infection. The current study could confirm that the rate of low-grade infection is relevant among femoral shaft nonunion and can be sufficiently detected after long-term culturing. This is in line with other studies demonstrating the importance of long-term culturing in contrast to short-term culturing (Table 5) [59,60,61,62,63].

In addition, in accordance with our study, the rate of septic nonunion in patients with presumed aseptic nonunion is indicated between 0% to 37%. However, patients, regardless of the location of the nonunion and the type of initial fracture stabilization, were included [56]. Thus, it is even more interesting that patients treated by a soft tissue-preserving procedure as intramedullary nailing is assumed, presented such a high rate of positive bacterial cultures. To our knowledge, there are no further studies that provide an explanation for this: Possible reasons for up to 43% of positive bacterial cultures might be either a disturbed skin barrier because of the contusion during the initial trauma, difficulties in the initial fracture stabilization with damage to the soft tissue due to the fact that the majority of the included patients were secondary transferred to our Level I trauma center or a secondary hematogenous colonization of the atrophic nonunion area.

In contrast, a clinically relevant cut-off value for preoperative systemic inflammation markers (C-reactive protein, white blood cell count) could not be observed in the current patient cohort with unsuspected proof of bacteria, which is consistent with a study performed by Wang et al. that laboratory analysis of serum inflammatory markers is not an effective screening method for septic nonunion [64]. Thus, we cannot recommend ruling out the possibility of nonunion-caused low-grade infection preoperatively by a sole consideration of CRP values or WBC.

Next, this study highlighted the objective and subjective long-term clinical outcomes. Interestingly, there was no significant difference regarding the number of patients with additional further interventions following positive bacterial growth detected during short-term culturing compared to those with only positive long-term culturing. Furthermore, clinically meaningful parameters resulting in a statistically reliable statement on whether or not additional surgical procedures are mandatory, following reamed exchange nailing in all cases of septic femoral shaft nonunion with unsuspected proof of bacteria, could not be found. Regarding the objective outcome represented by the LEFS at least one year after the final surgical revision, statistically significant differences were not detected after osseous healing in both groups. These results confirm the available literature [22,65,66]. In contrast, the PCS of SF-12—as a tool for assessing physical functioning and pain—demonstrated a significantly worse outcome in the case of low-grade infection compared to the aseptic femoral shaft nonunion, with the values themselves being comparable to the current literature [67]. The finding is noteworthy because a subsequent surgical intervention does not significantly impact the Physical Component Summary of SF-12. This indicates that low-grade infection alone—even after complete osseous healing of the nonunion—has an effect on the outcome similar to fracture-related infection [68,69], which may be caused by chronic inflammation—although a significant increase in the acute-phase protein CRP was not detected in the current study—and highlights the importance of also addressing low-grade infected nonunion early on to achieve optimal outcomes.

In addition to the multifactorial cause of impaired fracture healing [36], there is also the complicating fact that with the currently available diagnostic methods, the reliable exclusion of germ detection is only possible by intraoperative sample collection—implicating that an additional surgical procedure seems to be necessary to gain samples for microbiology and histology diagnostics before the actual nonunion revision. This is why both the single-stage and the two-stage surgical procedure, including adequate sample collection for microbiological diagnostics in the first step and surgical nonunion revision in the second step, are reported to be sufficient options in the recent literature [70], with previous studies demonstrated that the positive evidence of germs in a single-stage procedure does not generally result in treatment failure [22,51]. Nevertheless, a surgical procedure in septic nonunion differs in part significantly from the surgical revision of an aseptic nonunion, due to the need to address the infection and resultant biofilm formation in addition to the “singular” failure of the bone to unite in aseptic nonunion, which is why the authors propose the following procedure: If the preoperative patient’s history, as well as the clinical, laboratory and radiological examination, reveal indications of a possible underlying infectious event, further surgical revision is performed in the sense of a two-stage procedure with surgical specimen collection prior to definitive nonunion revision. Only if there is no indication for the presence of septic nonunion, the single-stage procedure is suggested. In this case, however, empirical antibiotic therapy should be initiated at the end of surgical nonunion revision until complete microbiological and histological diagnostics are obtained, while the frequency of intraoperative bacterial detection is relevant, even in the absence of preoperative signs of infection. In case of low-grade infected nonunion, following chronic fracture-related infection or periprosthetic infection, adjuvant test-appropriate systemic antibiotic therapy should be applied in addition to surgical therapy [71,72]. When a septic femoral shaft nonunion is present, there is no pressure to bring about an immediate definitive surgical treatment solution at any cost. Rather, the greatest possible care should be taken to optimize the patient prior to the surgical revision procedure. The main goal is to identify and treat potential risk factors that could delay or completely compromise nonunion healing.

Limitations of this study inherently include the retrospective study design. To our knowledge, this is one of only a few studies that focused exclusively on femur diaphysis using routine clinical diagnostics to demonstrate that the presence of unexpected evidence of bacteria has a relevant impact on daily clinical practice. The strength of the study is the large number of patients treated by the same surgical team at the same institution using a standardized treatment protocol.

## 5. Conclusions

The diagnosis of low-grade infection in femoral shaft nonunion remains challenging using routine clinical diagnostics such as preoperative systemic inflammatory markers or common risk factors because, despite an open soft tissue injury, no tools used in daily clinical practice could be identified for diagnosing low-grade infection. This is even more important since a worse subjective outcome in terms of physical function and pain has been observed in the case of low-grade infection—even after complete osseous healing of the femoral shaft nonunion. Furthermore, the probability of additional surgical interventions after the single-step procedure to achieve complete osseous healing is higher in cases of low-grade infected nonunion of the femoral shaft compared to aseptic femoral shaft nonunion.

## Figures and Tables

**Table 1:** Patients’ data overview. Values are presented as mean standard deviation or as total number of patients.

Parameter	Number
**Gender**	
Male	45
Female	13
**Age**	46.3 ± 2.1 (range 18–81) years
**Fracture location**	
Proximal part of the femoral shaft	19
Middle part of the femoral shaft	27
Distal part of the femoral shaft	12
**Fracture pattern according to the AO/OTA classification ^1^**	
Type A1	16
Type A2	17
Type A3	7
Type B1	3
Type B2	5
Type B3	3
Type C1	1
Type C2	3
Type C3	3
**Initial soft tissue injury**	
Closed fracture	50
Gustilo–Anderson open fracture classification I–III	8
**Nonunion type**	
Hypertrophic	40
Atrophic/Oligotrophic	18
**Comorbidities**	
Charlson comorbidity index	0.3 ± 0.1 (range 0–3) points
Nicotine abuse	12
Diabetes mellitus	6
**Period of time between initial fracture fixation and nonunion revision**	11.2 ± 1.0 (range 4–32) months

^1^ AO Foundation/Orthopaedic Trauma Association.

**Table 2:** Breakdown of organisms cultured.

Organism	Number of Isolates (Total *n* = 29)
Coagulase-negative *Staphylococcus* spp.	
*Staphylococcus epidermidis*	10
*Staphylococcus capitis*	3
*Staphylococcus lugdunensis*	3
*Staphylococcus haemolyticus*	2
*Staphylococcus warneri*	2
*Staphylococcus hominis*	1
*Staphylococcus aureus*	1
*Streptococcus alactolyticus*	1
*Enterococcus faecalis*	2
*Pseudomonas aeruginosa*	1
*Pseudomonas fluorescenses*	1
*Cutibacterium acnes*	2

**Table 3:** Evaluation of injury-related risk factors for the occurrence of positive bacterial cultures and/or nonunion. Values are presented as total number of patients.

Parameter	Group P (Positive Cultures)	Group N (Negative Cultures)	*p*-Value
**Fracture location**			
Proximal part of the femoral shaft	7	12	
Middle part of the femoral shaft	11	16	
Distal part of the femoral shaft	7	5	0.472
**Fracture pattern according to the AO/OTA classification**			
Type A	19	21	
Type B	1	10	
Type C	5	2	0.068
**Initial soft tissue injury**			
Closed fracture	19	31	
Gustilo–Anderson open fracture I–III	6	2	0.045
**Nonunion type**			
Hypertrophic	15	25	
Atrophic/Oligotrophic	10	8	0.199

**Table 4:** Overview of the objective and subjective outcome at least one year after exchange nailing procedure. Values are presented as mean standard deviation.

Test Procedure	Group P (Positive Cultures)	Group N (Negative Cultures)	*p*-Value
LEFS	46.0 ± 5.1 points	51.6 ± 5.7 points	0.479
PCS of SF-12	35.6 ± 3.1 points	44.4 ± 2.6 points	0.040
MCS of SF-12	49.5 ± 3.2 points	50.1 ± 2.4 points	0.875

LEFS: best functional outcome with 80 points; SF-12: best possible outcome with 100 points.

**Table 5:** Literature overview of intraoperative germ detection in nonunion revisions in regard to the microbiological diagnostics.

Study	Inclusion Criteria	Number of Patients	Bacterial Detection Rate	
Gille et al. [62]	preoperatively aseptic classified tibial shaft nonunion	23	culturing for 14 days:	0%
Olszewski et al. [59]	nonunion without signs of infection but with risk factors for infection	453	culturing for 5 days:	20%
Dapunt et al. [60]	atrophic nonunion of long bones (32.7% with clinical signs of infection)	49	culturing for 2 days:	6.8%
			culturing for 5 days:	10.2%
			sonication and culturing for 14 days:	57.1%
Palmer et al. [61]	nonunion of long bones	34	culturing for 5 days:	23.5%

## Data Availability

The data presented in this study are available on request from the corresponding author. The data are not publicly available due to privacy.
